# Thymosin β 10 is overexpressed and associated with unfavorable prognosis in hepatocellular carcinoma

**DOI:** 10.1042/BSR20182355

**Published:** 2019-03-15

**Authors:** Chunrong Song, Zhong Su, Jing Guo

**Affiliations:** 1 Liver Diseases Branch, Affiliated Hospital of Shaanxi University of Traditional Chinese Medicine, Xianyang, Shaanxi 712000, China; 2 Department of Oncology, People’s Hospital of Zouping City, Binzhou, Shandong 256200, China; 3 Clinical Laboratory, Xi’an Central Hospital Affiliated to Xi’an Jiaotong University, Xi’an, Shaanxi 710003, China

**Keywords:** biomarker, hepatocellular carcinoma, liver cancer, TMSB10

## Abstract

Thymosin β 10 (TMSB10) has been demonstrated to be overexpressed and function as an oncogene in most types of human cancer including hepatocellular carcinoma (HCC). In our study, we present more evidence about the clinical significance and biological function of TMSB10 in HCC. First, we observed levels of TMSB10 expression were obviously increased in HCC tissues compared with normal liver tissues at The Cancer Genome Atlas (TCGA) datasets. Furthermore, we confirmed that *TMSB10* mRNA and protein levels were also increased in HCC tissue samples compared with normal adjacent normal liver tissue samples. In addition, we found high TMSB10 expression was remarkably associated with the advanced tumor stage, large tumor size, distant metastasis, and poor prognosis, and acted as an independent factor for predicting poor overall survival in HCC patients. Loss-of-function studies suggested silencing of TMSB10 expression dramatically reduced cell proliferation, migration, and invasion in HCC. In conclusion, TMSB10 may hold promise as a tumor biomarker for predicting prognosis and a potential target for developing a novel therapeutic strategy.

## Introduction

Liver cancer is the seventh most common cancer worldwide accounting for 841080 new diagnosed cases in 2018 [1]. Meanwhile, liver cancer is the third leading cause of cancer-related deaths worldwide with an estimated 781631 deaths in 2018 [1]. Hepatocellular carcinoma (HCC) is a major type of primary liver cancer, and represents 85–90% of all liver cancer patients [2]. In China, chronic hepatitis B virus (HBV) infection is the strongest risk factor of HCC, and accounts for the vast majority of HCC [3]. Despite recent therapeutic advances in interventional therapy and targetted therapy, the prognosis of HCC is still unfavorable with overall 5-year survival rates as low as 11–30% [4]. Intrahepatic metastasis and tumor recurrence are the major reason of treatment failure in HCC cases [5]. Therefore, investigating the pathogenesis of HCC is particularly important for identifying credible biomarkers and effective therapeutic targets.

Thymosin β 10 (TMSB10) is a member of the family of β-thymosins, which contain highly conserved acidic 5-kDa peptides consisting of 40–44 amino acid residues [6]. TMSB10 consists of 43 amino acid residues and is mainly localized in cytoplasm [7]. TMSB10 has been demonstrated to be overexpressed in most types of human cancer, and participate in regulating cell proliferation and motility [7]. In HCC, TMSB10 originally showed high reactivity rate with 96% in tumor samples [8]. Then, TMSB10 was found to be overexpressed in HCC tissues, and correlated with TNM stage and overall survival in HCC patients [9]. Our study presents more evidence about the relationship between TMSB10 and HCC. In our study, we explore the clinical and prognostic significance of TMSB10 in HCC through analyzing the association between TMSB10 and clinical parameters of HCC patients. Moreover, we conducted loss-of-function studies to investigate the impact of TMSB10 on cell proliferation, migration, and invasion in HCC.

## Materials and methods

### Human HCC tissue samples

The experiments were approved by the Ethics Committee of Affiliated Hospital of Shaanxi University of Traditional Chinese Medicine, People’s Hospital of Zouping City and Xi’an Central Hospital Affiliated to Xi’an Jiaotong University, and performed according to the ethical principles of Declaration of Helsinki. All clinical tissue samples in the present study were obtained from patients with informed consents at Affiliated Hospital of Shaanxi University of Traditional Chinese Medicine, People’s Hospital of Zouping City or Xi’an Central Hospital Affiliated to Xi’an Jiaotong University. A total of 102 paraffin-embedded HCC tissue samples and 30 normal adjacent normal liver tissue samples were collected for the TMSB10 immunohistochemistry staining. Thirty pairs of fresh HCC tissues and adjacent normal liver tissues were collected for detecting *TMSB10* mRNA expression. The pathologic diagnosis of all samples was independently conducted by two pathologists. The system treatment of all cases was according to the Chinese Clinical Guideline for HCC. Clinical data included the patient gender, age, tumor stage, tumor size, vascular invasion, metastasis, HBV infection, histological differentiation, and follow-up.

### Cell lines

The human liver cancer cell lines (HepG2 and Huh-7) and human normal liver cell (WRL68 and LO-2) were obtained from the Cell Bank of Chinese Academy of Sciences and cultured in complete growth medium as recommended by the manufacturer.

### RNA extraction and quantitative real-time PCR

Total RNA was isolated from cells or tissues by using RNAiso Plus (Takara, Dalian, China) in accordance with the manufacturer’s protocol. RNA reverse transcription was performed by using PrimeScript RT Reagent Kit (Takara, Dalian, China), and quantitative real-time PCR (qRT-PCR) was conducted by TB Green Premix ExTaq II (Takara, Dalian, China) at LightCycler 480 (Roche Applied Science, Indianapolis, IN, U.S.A.). Glyceraldehyde-3-phosphate dehydrogenase (GAPDH) was measured as the internal control. The following primer pairs were used for qRT-PCR: 5′-TGGCAGACAAACCAGACATGG-3′ (Forward) and 5′-CGAAGAGGACGGGGGTAGG-3′ (Reverse) for TMSB10; 5′-CCCATCACCATCTTCCAGGAG-3′ (Forward) and 5′-GTTGTCATGGATGACCTTGGC-3′ (Reverse) for GAPDH.

### Immunohistochemistry

 Tissue sections (3 μm) were deparafinized in xylene and rehydrated by passing through a graded series (of alcohol) of 100, 95, 90, 80, and 70% ethanol. Then, 0.3% H_2_O_2_ was used to blocked endogenous peroxidase at room temperature for 10 min. For antigen retrieval, the tissue sections were boiled in EDTA (1 mM, pH 8.0) for 15 min. Sections were then incubated with anti-TMSB10 antibody (1:250 dilution; Abcam, Cambridge, U.K.) at 4°C overnight. After three washes in PBS, sections were incubated with horseradish peroxidase–conjugated secondary antibody, and colored with 3,3′-diaminobenzidine (DAB) and Hematoxylin.

TMSB10 staining was scored independently by two pathologists who were blinded to the clinical data. The proportion of positive tumor cell was scored as: 0, 0–9% positive tumor cells; 1, 10–25% positive tumor cells; 2, 26–50% positive tumor cell; 3, >50% positive tumor cell. Staining intensity was scored as: 0, no staining; 1, weak staining; 2, moderate staining; and 3, strong staining, as described [9]. The final scores ranged from 0 to 12. Based on the published study [9], a score of ≥4 was defined as high TMSB10 expression group and scores of <4 defined low TMSB10 expression group.

### Cell transfection

Knockdown of endogenous TMSB10 was performed by siRNA-TMSB10 (si-TMSB10), which was synthesized by Shanghai Integrated Biotech Solutions Co., Ltd. (Shanghai, China). Briefly, HCC cells were seeded in six-well plate. At a density of 70% confluence, HCC cells were transfected with si-TMSB10 or si-NC by using the Lipofectamine RNAimax reagent (Invitrogen, Carlsbad, CA, U.S.A.) in accordance with the protocol provided by the manufacturer.

### Cell proliferation assay

The Cell Counting Kit–8 assay (CCK–8, Dojindo Molecular Technologies, Kumamoto, Japan) was used to assess cell proliferation ability. Briefly, transfected HepG2 and Huh-7 cells were plated in 96–well plates with 3000 cells/well. After 24, 72, and 120 h incubation, 10 μl of CCK–8 solution was added to each well for 2 h at 37°C. The absorbance was measured at 450 nm using a spectrophotometer.

### Cell migration assay

Transwell chamber (8 μm pore size; Corning, NY, U.S.A.) in 24-well plate was used for cell migration assay. Briefly, transfected HepG2 and Huh-7 cells were resuspended in 200 μl culture medium without FBS at a density of 5 × 10^4^ cells/well, and seeded into the upper chamber. The bottom chamber of transwell plates was supplemented with 500 μl culture medium with 20% FBS as a chemoattractant. After 18 h incubation, cells on the lower surface were fixed with polyoxymethylene, and stained with 0.1% Crystal Violet staining solution. The cells on the bottom of the membrane were calculated from five random light microscopic fields.

### Cell invasion assay

Transwell chamber and matrigel matrix (BD Biosciences, Franklin Lakes, NJ, U.S.A.) were used for cell invasion assay. Briefly, the transwell membrane was precoated with 50 μl matrigel matrix. The other procedures were similar to the migration assay.

### Statistical analysis

All statistical analyses were performed using SPSS software (version 17.0; Chicago, IL, U.S.A.). Comparisons between two groups were done using the Student’s *t* test for continuous data and the chi-square test for categorical data. The chi-square test was used to estimate the association between TMSB 10 expression and clinicopathological characteristics of HCC patients. Survival analysis was calculated by Kaplan–Meier method and compared by log-rank test. The prognostic varieties were evaluated by univariate and multivariate Cox proportional hazards regression analyses. A significant difference was considered when *P*<0.05.

## Results

### TMSB10 expression is increased in HCC

**Figure 1:**
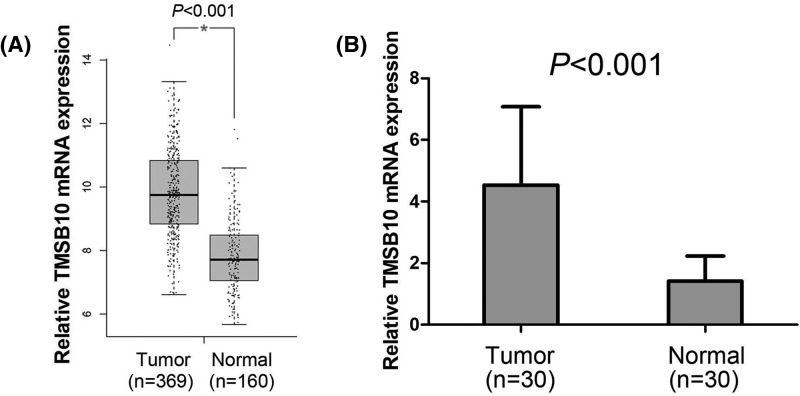
TMSB10 expression is increased in HCC (**A**) TMSB10 expression was increased in HCC tissues compared with normal liver tissues. (**B**) *TMSB10* mRNA levels were elevated in HCC tissues compared with paired adjacent normal liver tissues.

**Figure 2:**
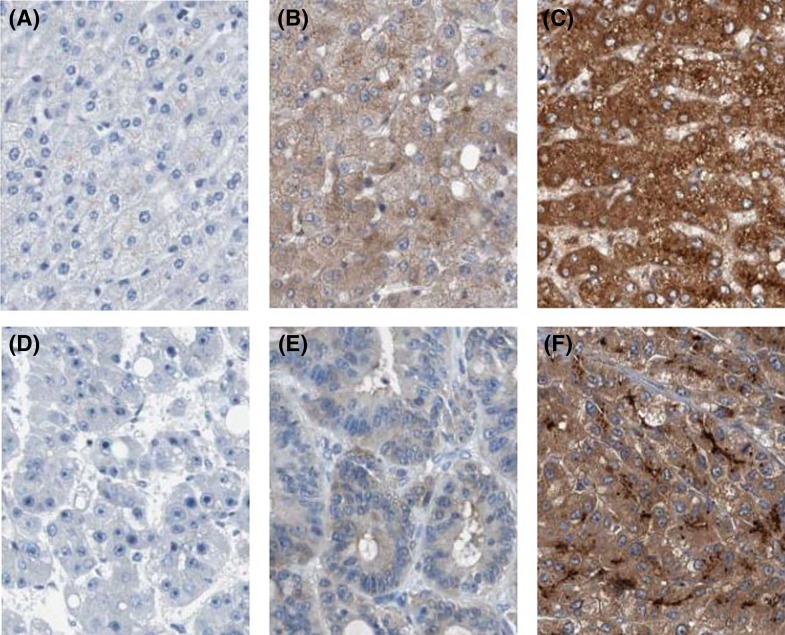
Immunohistochemical staining of TMSB10 (**A**) Negative expression of TMSB10 in normal liver tissue. (**B**) Low expression of TMSB10 in normal liver tissue. (**C**) High expression of TMSB10 in normal liver tissue. (**D**) Negative expression of TMSB10 in HCC tissue. (**E**) Low expression of TMSB10 in HCC tissue. (**F**) High expression of TMSB10 in HCC tissue.

First, we observed the TMSB10 expression levels in HCC tissues and normal liver tissues from The Cancer Genome Atlas (TCGA) datasets, and found levels of TMSB10 expression were obviously increased in HCC tissues compared with normal liver tissues (*P*<0.001, Figure 1A). Furthermore, *TMSB10* mRNA expression was measured by qRT-PCR in 30 pairs of fresh HCC tissues and adjacent normal liver tissues. We found the overall fold increase in *TMSB10* mRNA levels in HCC tissues was 3.21-times greater than that in the paired adjacent normal liver tissues (*P*<0.001, Figure 1B). Then, we detected TMSB10 protein expression by immunohistochemistry in 102 paraffin-embedded HCC tissue samples and 30 normal adjacent normal liver tissue samples (Figure 2A–F). We observed that The TMSB10 protein expression was markedly higher in 65 (63.73%) HCC tissue samples compared with the normal adjacent normal liver tissue samples (36.67%) (*P*=0.008, Table 1).

**Table 1:** TMSB10 protein expression between HCC tissues and normal liver tissues

	Number	TMSB10		***P***
		High expression	Low expression	
Normal liver tissues	30	11	19	0.008
HCC tissues	102	65	37	

### High TMSB10 expression is correlated with the malignant status in HCC patients

As shown in Figure 2D–F, TMSB10 immunohistochemical staining was observed only in the cytoplasm of tumor cells. For investigating the clinical value of TMSB10 expression in HCC patients, the associations between TMSB10 expression and clinicopathological characteristics of HCC patients were analyzed by the chi-square test, as shown in Table 2. The high TMSB10 expression was remarkably associated with the advanced tumor stage (I–II compared with III–IV, *P*=0.005), tumor size (<5 compared with ≥5 cm, *P*=0.028), and distant metastasis (Absent compared with Present, *P*=0.001), but was not associated with age (*P*=0.714), gender (*P*=0.205), HBV infection (*P*=0.159) and histological differentiation (*P*=0.294).

**Table 2:** Associations between TMSB10 expression and clinicopathological features in HCC

Characteristics	*n*	High expression	Low expression	*P*
Age (years)				
<50	41	27	14	0.714
≥50	61	38	23	
Gander				
Female	36	20	16	0.205
Male	66	45	21	
Tumor stage				
I–II	32	14	18	0.005
III–IV	70	51	19	
Tumor size				
<5 cm	60	33	27	0.028
≥5 cm	42	32	10	
Metastasis				
Absent	85	48	37	0.001
Present	17	17	0	
HBV infection				
Absent	30	16	14	0.159
Present	72	49	23	
Histological differentiation				
Well	62	42	20	0.294
Moderate/Poor	40	23	17	

### High TMSB10 expression is correlated with short overall survival time in HCC patients

**Figure 3:**
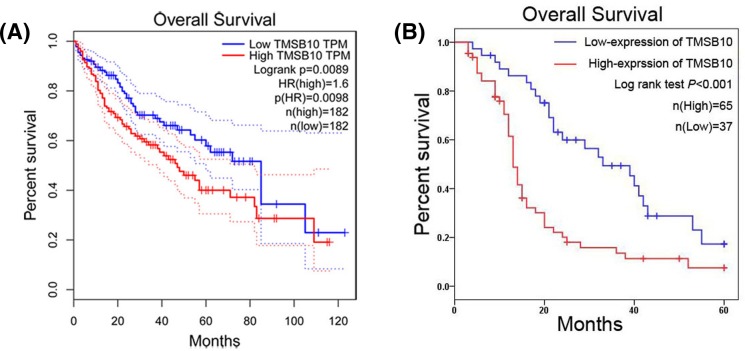
High TMSB10 expression is correlated with short overall survival time in HCC patients (**A**) Survival curves of the HCC patients with high and low TMSB10 expression from TCGA datasets. (**B**) Survival curves of the HCC patients with high and low TMSB10 expression from our study.

The association between TMSB10 expression and overall survival was analyzed in HCC patients from TCGA datasets. We observed HCC patients with high expression of TMSB10 had shorter overall survival in comparison with HCC patients with low expression of TMSB10 (*P*=0.009, Figure 3A). Furthermore, our result of Kaplan–Meier survival analysis also suggested high TMSB10 expression was associated with short overall survival time in HCC patients (*P*<0.001, Figure 3B), which was consistent with the result of TCGA datasets. Moreover, we identified tumor stage (*P*=0.009), tumor size (*P*=0.039), distant metastasis (*P*<0.001), and TMSB10 expression (*P*<0.001) as prognostic factors for overall survival in HCC patients through univariate Cox proportional hazards regression analysis (Table 3). Then, multivariate Cox proportional hazards regression analysis indicated that high expression of TMSB10 was an independent factor for predicting poor overall survival in HCC patients (*P*=0.044, Table 3).

**Table 3:** Univariate and multivariate Cox regression analysis for overall survival in HCC patients

Parameter	Univariate analysis			Multivariate analysis		
	HR	95% CI	*P*	HR	95% CI	*P*
Age (years)						
(<50 compared with ≥50)	0.692	0.433–1.104	0.122			
Gender						
(Female compared with Male)	1.418	0.868–2.317	0.163			
Tumor stage						
(I–II compared with III–IV)	2.015	1.191–3.407	0.009	1.111	0.525–2.350	0.784
Tumor size						
(<5 compared with ≥5 cm)	1.626	1.024–2.582	0.039	1.020	0.580–1.793	0.946
Metastasis						
(Absent compared with Present)	4.995	2.666–9.358	<0.001	3.622	1.859–7.054	<0.001
HBV infection						
(Absent compared with Present)	1.547	0.912–2.624	0.105			
Histological differentiation						
(Well compared with Moderate/Poor)	1.197	0.752–1.907	0.449			
TMSB10 expression						
(Low compared with High)	2.545	1.545–4.191	<0.001	1.949	1.018–3.733	0.044

Abbreviations: HR, hazard ratio; 95% CI, 95% confidence interval.

### TMSB10 functions as an oncogene to regulate cell proliferation, migration, and invasion

**Figure 4:**
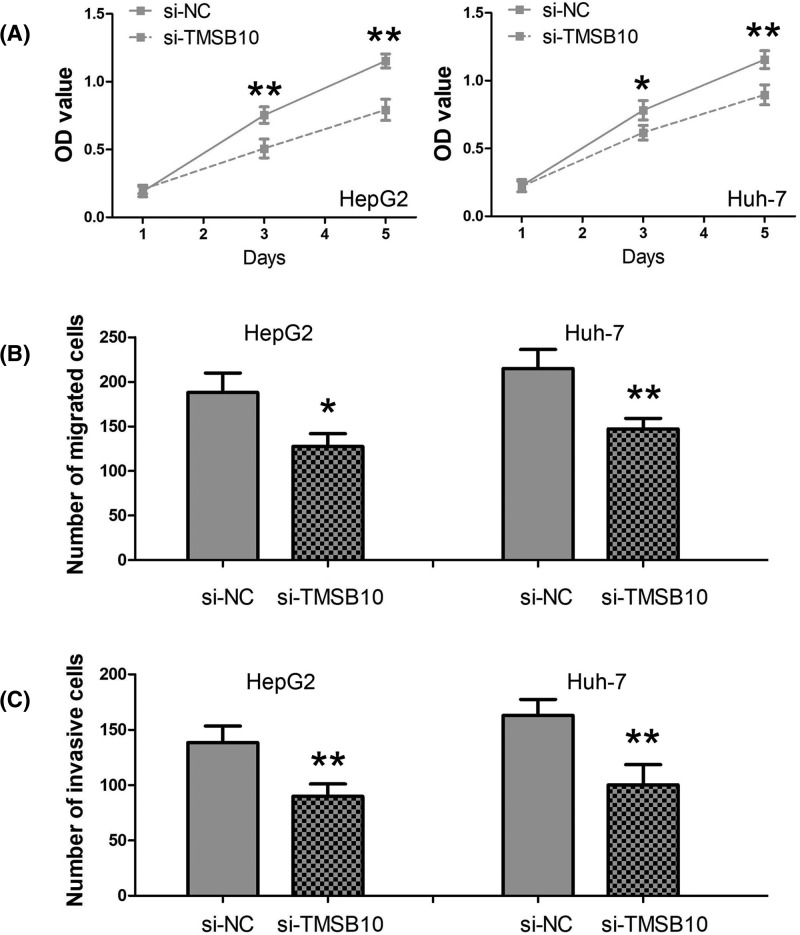
The impact of TMSB10 on HCC cell proliferation, migration, and invasion (**A**) Silencing of TMSB10 expression conspicuously inhibited cell viability of HepG2 and Huh-7 cells at 72 and 120 h. (**B**) Silencing of TMSB10 expression reduced the migration ability of HepG2 and Huh-7 cells. (**C**) Silencing of TMSB10 expression decreased the invasion ability of HepG2 and Huh-7 cells (*, *P*<0.01; **, *P*<0.001).

To explore the biological roles of TMSB10 in HepG2 and Huh-7 cells, we endogenously knocked down TMSB10 expression in HepG2 and Huh-7 cells by si-TMSB10. We investigated the impact of TMSB10 on cell proliferation by CCK–8 assay, and found silencing of TMSB10 expression conspicuously inhibited cell viability of HepG2 and Huh-7 cells at 72 and 120 h (*P*<0.01; Figure 4A). We next explored the impact of TMSB10 on cell migration and invasion abilities by transwell cell migration and invasion assays, and found silencing of TMSB10 expression dramatically reduced the migration and invasion abilities of HepG2 and Huh-7 cells (*P*<0.01; Figure 4B,C).

## Discussion

TMSB10 is a member of the family of β-thymosins, and has been found to be overexpressed in most types of human cancers including liver cancer [8,9], gastric cancer [10], pancreatic cancer [11,12], cholangiocarcinoma [13], renal cell carcinoma [14], ovarian cancer [15,16], lung cancer [17,18], breast cancer [19,20], and thyroid cancer [21–23]. Besides, Santelli et al. [24] suggested that high TMSB10 expression levels were observed in human colon cancer, goniolma, breast cancer, ovarian cancer, uterine carcinoma, esophageal cancer cell lines. In our study, we presented more evidence about TMSB10 expression status in HCC. First, we observed the TMSB10 expression levels in HCC tissues and normal liver tissues from TCGA datasets, and found levels of TMSB10 expression were obviously increased in HCC tissues compared with normal liver tissues. Furthermore, we confirmed that *TMSB10* mRNA and protein levels were also increased in HCC tissue samples compared with normal adjacent normal liver tissue samples, which was consistent with Theunissen et al.’s [8] and Wang et al.’s [9] reports. Furthermore, we investigated the clinical value of TMSB10 expression in HCC through analyzing the associations between TMSB10 expression and clinicopathological characteristics, and found that high TMSB10 expression was remarkably associated with the advanced tumor stage, tumor size, and distant metastasis in HCC patients. Similarly, Wang et al. [9] also showed that patients with advanced TNM stage had higher level of TMSB10 expression than those with early TNM stage. Moreover, high TMSB10 expression was found to be associated with advanced clinical stage, lymph node metastasis, distant metastases, poor degree of differentiation, positive vascular endothelial growth factor, and positive vascular endothelial growth factor-C expression in non-small cell lung cancer patients [25,26]. In breast cancer patients, Bouchal et al. [27] indicated that high levels of *TMSB10* mRNA and protein were both associated with lymph node metastasis and unfavorable histological grade. In addition, Zhang et al. [28] showed that TMSB10 overexpression was correlated with cervical and central neck lymph node metastasis in patients with papillary thyroid carcinoma.

We next explored the prognostic significance of TMSB10 expression in HCC. We analyzed the association between TMSB10 expression and overall survival of HCC patients in TCGA datasets, and found HCC patients with high expression of TMSB10 had shorter overall survival in comparison with HCC patients with low expression of TMSB10. Furthermore, our survival analysis suggested high TMSB10 expression was associated with short overall survival time in HCC patients, and acted as an independent factor for predicting poor overall survival in HCC patients. Similarly, Wang et al. [9] also showed high TMSB10 expression was an independent prognostic factor for both overall survival and disease-free survival in HCC patients. In addition, Gu et al. [25] suggested TMSB10 overexpression predicted unfavorable postoperative survival in non-small cell lung cancer patients. In breast cancer patients, increased TMSB10 expression was suggested to be correlated with short metastasis-free survival, relapse-free survival, and overall survival [27,29]. Besides, TMSB10 served as a member of gene expression signature for predicting survival in cervical carcinoma patients [30] and metastatic melanoma patients [31].

The biological function of TMSB10 in HCC cells was still unknown. For investigating the impact of TMSB10 on HCC cell proliferation, migration, and invasion, we endogenously knocked down endogenous expression in HCC cells by si-TMSB10. We found silencing of TMSB10 expression dramatically reduced the proliferation, migration, and invasion abilities of HCC cells. Moreover, Zhang et al. [29] reported knocking down TMSB10 expression mediated AKT/FOXO signaling to depress breast cancer cell proliferation, invasion, and migration *in vitro* and *in vivo*. In lung adenocarcinoma cells, Li et al. [32] revealed that TMSB10 promoted cell proliferation, and arrested cell cycle at S-phase and G_2_/M-phase. However, Sribenja et al. [13] found down-regulation of TMSB10 expression obviously promotes cholangiocarcinoma cell migration and invasion *in vitro*, and tumor metastasis *in vivo*. The discrepancy between our data and Sribenja et al.’s [13] data would be most likely due to tumor heterogeneity.

In conclusion, TMSB10 expression is relatively high in HCC tissues, and correlated with the advanced tumor stage and distant metastasis in HCC patients. High expression of TMSB10 is an independent factor for predicting poor overall survival in HCC patients. Loss of TMSB10 expression reduces the proliferation, migration, and invasion abilities of HCC cells.

## Abbreviations

AKT: serine/threonine kinase 1
CCK-8: cell counting kit–8
FOXO: forkhead box O
GAPDH: glyceraldehyde-3-phosphate dehydrogenase
HBV: hepatitis B virus
HCC: hepatocellular carcinoma
qRT-PCR: quantitative real-time PCR
TMSB10: Thymosin β 10
TCGA: The Cancer Genome Atlas
TNM: tumor node metastasis
